# Correlations Between the Characteristics of Alternative Splicing Events, Prognosis, and the Immune Microenvironment in Breast Cancer

**DOI:** 10.3389/fgene.2021.686298

**Published:** 2021-06-14

**Authors:** Youyuan Deng, Hongjun Zhao, Lifen Ye, Zhiya Hu, Kun Fang, Jianguo Wang

**Affiliations:** ^1^Department of General Surgery, Xiangtan Central Hospital, Xiangtan, China; ^2^Department of Pharmacy, Third Hospital of Changsha, Changsha, China; ^3^Department of Surgery, Yinchuan Maternal and Child Health Hospital, Yinchuan, China

**Keywords:** breast cancer, alternative splicing, immune cell infiltration, the cancer genome atlas, splicing factor

## Abstract

**Objective:**

Alternative splicing (AS) is the mechanism by which a few genes encode numerous proteins, and it redefines the concept of gene expression regulation. Recent studies showed that dysregulation of AS was an important cause of tumorigenesis and microenvironment formation. Therefore, we performed a systematic analysis to examine the role of AS in breast cancer (Breast Cancer, BrCa) progression.

**Methods:**

The present study included 993 BrCa patients from The Cancer Genome Atlas (TCGA) database in the genome-wide analysis of AS events. We used differential and prognostic analyses and found differentially expressed alternative splicing (DEAS) events and independent prognostic factors related to patients’ overall survival (OS) and disease-free survival (DFS). We divided the patients into two groups based on these AS events and analyzed their clinical features, molecular subtyping and immune characteristics. We also constructed a splicing factor (SF) regulation network for key AS events and verified the existence of AS events in tissue samples using real-time quantitative PCR.

**Results:**

A total of 678 AS events were identified as differentially expressed, of which 13 and 10 AS events were independent prognostic factors of patients’ OS and DFS, respectively. Unsupervised clustering analysis based on these prognostic factors indicated that the Cluster 1 group had a better prognosis and more immune cell infiltration. SFs were significantly related to the expression of AS events, and AA-RPS21 was significantly upregulated in tumors.

**Conclusion:**

Alternative splicing expands the mechanism of breast cancer progression from a new perspective. Notably, alternative splicing may affect the patient’s prognosis by affecting the infiltration of immune cells. Our research provides important guidance for subsequent studies of AS in breast cancer.

## Introduction

Breast cancer (BrCa) is the most common malignant tumor in women. There were approximately 2.1 million newly diagnosed BrCa cases worldwide in 2018, which accounts for approximately 25% of the total number of female malignancies and poses a serious threat to women’s health and a heavy burden to public health (Schneider et al., 2014; Bray et al., 2018). Early diagnosis significantly improves the survival rate of patients. The 5-year survival rate of patients diagnosed with BrCa before metastasis is 99% (Siegel et al., 2018), and the 5-year survival rate of patients whose tumor has spread to distant organs is only 26% (Koual et al., 2019). Due to the heterogeneity and complexity of BrCa, traditional inspection methods, such as immunohistochemical testing, do not identify effective biomarkers to screen and evaluate BrCa (Network, 2012). Researchers examined the mechanisms of the occurrence and development of BrCa from various aspects, such as gene expression disorders (Cruz et al., 2018), copy number variation (Long et al., 2018; Yang et al., 2019) and DNA methylation (Kresovich et al., 2019; Yari and Rahimi, 2019). Although these studies achieved promising results, they were primarily limited to the transcriptional level, and the post-transcriptional level, such as alternative splicing (AS), was neglected.

AS is an important way to generate greater transcriptome and proteomic diversity in a limited genome (Cieply and Carstens, 2015). Pre-mRNAs may be spliced into mature mRNAs by retaining specific intron regions or excluding specific exons in multi-exon genes (Kelemen et al., 2013), which generates structural and functional protein variants, and further promotes protein diversity and phenotypic complexity (Leoni et al., 2011). The genetic similarity between human and chimpanzee DNA is 99%, but the homology of mature mRNA is less than 60% (Barbosa-Morais et al., 2012). Genome-wide studies show that 92–95% of human exons have undergone alternative splicing (Feng et al., 2013), and the expression of most cellular transcripts have spatial and temporal differences (Wang et al., 2008). The importance of AS in the development of tumors was recognized recently (Srebrow and Kornblihtt, 2006). AS dysfunction leads to changes in the biological behavior of tumor cells, including cell proliferation (Endo, 2019), cell apoptosis (Pal et al., 2019), tumor angiogenesis (Pentheroudakis et al., 2019) and immune escape (Yao et al., 2016). Increasing evidence shows that the unbalanced expression or mis-expressed isomers of splicing variants is another feature of cancer (Ladomery, 2013). Therefore, cancer-specific splicing variants may be used as diagnostic, prognostic, and therapeutic targets. AS events were first reported as a prognostic biomarker for non-small cell lung cancer in 2017 (Li et al., 2017), and subsequent studies were performed in thyroid cancer (Lin et al., 2019), colorectal cancer (Xiong et al., 2018), pancreatic cancer (Yu et al., 2019) and other tumors. However, there are no related reports on the possibility of differentially expressed alternative splicing (DEAS) events as a biomarker for predicting the prognosis of BrCa.

The splicing of pre-mRNA is regulated by cis-acting elements and trans-acting factors. According to different positions and different effects on splicing sites, cis-acting elements are divided into exon splicing enhancers, exon splicing silencers, intron splicing enhancers and intron splicing silencers, which determine their affinity with homologous splicing factors (SFs). Trans-acting factors, including Ser/Arg-rich members and heterologous ribonucleoprotein family members, work by combining with exon splicing enhancers and silencers to further activate or inhibit specific splicing sites (Kornblihtt et al., 2013). According to their sequence, SFs affect the splicing site selection of the splicing regulatory complex (spliceosome) by binding to pre-mRNAs on exon splicing enhancers or silencers (Ule et al., 2006). Recent studies showed that abnormal AS events are caused by a maladjustment of SFs (Anczuków and Krainer, 2016). Therefore, the identification of splicing factors that are responsible for AS in breast cancer must be further studied.

The present study used the RNA sequence data from The Cancer Genome Atlas (TCGA) to perform a genome-wide systemic analysis of the AS events and SFs of BrCa. We combined the DEAS with clinical data to screen for biomarkers associated with survival and recurrence. Our results provide new insights and potential mechanisms for predicting the prognosis and evaluating clinical outcomes for BrCa patients.

## Materials and Methods

### Data Acquisition and Processing

We downloaded and integrated the RNA sequence data, gene expression data, methylation data and corresponding clinical information of BrCa patients from the TCGA data portal website (access date December 20, 2020^1^; Colaprico et al., 2016). To quantify the AS events for each BrCa patient, we used a Java application called SpliceSeq to explicitly quantify the splicing pattern and percent-spliced-in (PSI) for each AS event (Ryan et al., 2012). To generate a set of AS event data as reliably as possible, a strict filter condition of samples with PSI values ≥ 0.75, average PSI value ≥ 0.05 was used. The following inclusion criteria were used: (1) female; (2) patients diagnosed with BrCa using pathology; (3) the patient did not receive neoadjuvant therapy; (4) complete clinical characteristics, including age, histological classification, pathological stage, Stage T, Stage N, and stage M; (5) the patient survived at least 30 days after the surgery; and (6) corresponding mRNA splicing variant data were full-scale. A total of 993 patients were enrolled in our study cohort. For maximizing the value of the data and minimizing the possible bias caused by the deletion of the missing values, the IterativeImputer package in Python was used to perform multiple interpolations of the missing values (White et al., 2011). The UpSet diagram, created with UpSetR (version: 1.3.3), shows the interaction sets in 7 types of AS. Circos graphs were generated from Circlize (version 0.4.5) to visualize AS events and details of genes in chromosomes. The Pam50 subtype data of BrCa were obtained from Berger et al. (2018). We quantified the infiltration level of ssGSEA immune cell types in the R language Gsva package (Hänzelmann et al., 2013).

To accurately describe AS events, a unique annotation was assigned to each AS event by combining the splicing type, the ID number in the SpliceSeq database, and the corresponding gene symbol. For example, in the annotation “ME_HLCS_ID_96019,” the mutually exclusive exon (ME) represents the splicing type, ID_96019 represents the specific ID of the splicing variant, and HLCS is the corresponding genetic symbol.

### Identification and Functional Enrichment Analysis of DEAS Events

To identify DEAS between BrCa tissues and adjacent normal tissues, PSI values for each AS event were measured from the TCGA BrCa cohort (993 BrCa tissues and 113 adjacent normal tissues). Expression differences were characterized as difference multiples (log2FoldChange, log2FC) and adj.P value (Bonferroni corrected *P*-value). | log2FC | > 1 and adj.P < 0.05 indicate that the corresponding AS events were up-regulated or down-regulated, respectively. The heatmap and the volcano map were used to show the AS events expressed differently. The DEAS event parent genes were subjected to biological functional enrichment analysis to examine the potential mechanism of BrCa. The “Enrichplot” software package (version 1.6.0) of the R software (version 3.6.1) was used to perform the gene ontology (GO) and Kyoto Encyclopedia of Genes and Genomes (KEGG) analyses. A False Discovery Rate (FDR, *P*-value corrected by Benjamini-Hochberg method) less than 0.05 was considered significant.

### Building a Splicing Related Network

Seventy-one splicing factors (SFs) were verified in experiments, and these SFs belonged to two main families, Ser/ARG-rich (SR) protein and heteroribonucleoprotein (hnRNPs). The correlation between SF expression and the PSI value of DEAS events was analyzed (| R| > 0.5, *P* < 0.05), and the correlation graph was generated using Cytoscape (version 3.7.1).

### Investigating the Prognostic Value of DEAS Events

Based on the DEAS event, univariate Cox regression analysis and LASSO (Least Absolute selection Operator) regression analysis were performed on overall survival (OS) and disease-free survival (DFS) to determine independent prognostic factors in BrCa. The relationship between clinicopathological data and prognosis was further examined.

### Evaluation of the Correlation With Clinical, Molecular and Immune Characteristics

Based on the total independent risk factors, the “Consent ClusterPlus” package was used to classify the TCGA BrCa cohort in an unbiased and unsupervised manner (Wilkerson and Hayes, 2010; de Lena et al., 2017).

The following clustering settings were used in our study: maxK = 9; Clustering algorithm = PAM; and Correlation method = Euclidean. The relative change in area under the cumulative distribution function curve was used to determine the optimal clustering number K. Differences in clinicopathological information (T, N, M, and TNM stages), survival status (OS and DFS), immune cell content and molecular typing were analyzed between groups, and a violin diagram was used to visualize the different immune cell contents between different subgroups. We performed a survival analysis on the above-listed groups to verify the impact of these events on patient prognosis.

### Real-Time Fluorescence Quantitative PCR to Validate DEAS Events

The Medical Ethics Committee of Xiangtan Central Hospital approved the use of patient samples, and the study complied with the provisions of the Declaration of Helsinki (amended in 2013). Twenty cases of frozen BrCa tissue and paired adjacent tissue were collected from patients who received treatment at the General Surgery Department of Xiangtan Central Hospital from June 1, 2019 to December 1, 2019. Real-time fluorescence quantitative PCR was performed to verify DEAS events. Total RNA was isolated from frozen tissue using TRIzol (Invitrogen, ThermoFisher, CA, United States), and it was reverse transcribed into cDNA using the PrimeScript^TM^ RT kit (TaKaRa, Otsu, Japan). SYBR Premix Ex-Taq^TM^ (TaKaRa, Otsu, Japan) was performed in an FTB-3000P PCR system (Funglyn Biotech, Shanghai, China). The 2^–ΔΔ*CT*^ method was used to calculate the expression quantity of relative splicing variants. The corresponding PCR primers are listed in Supplementary Table 1.

## Results

### Overview of BrCa AS Events

**FIGURE 1:**
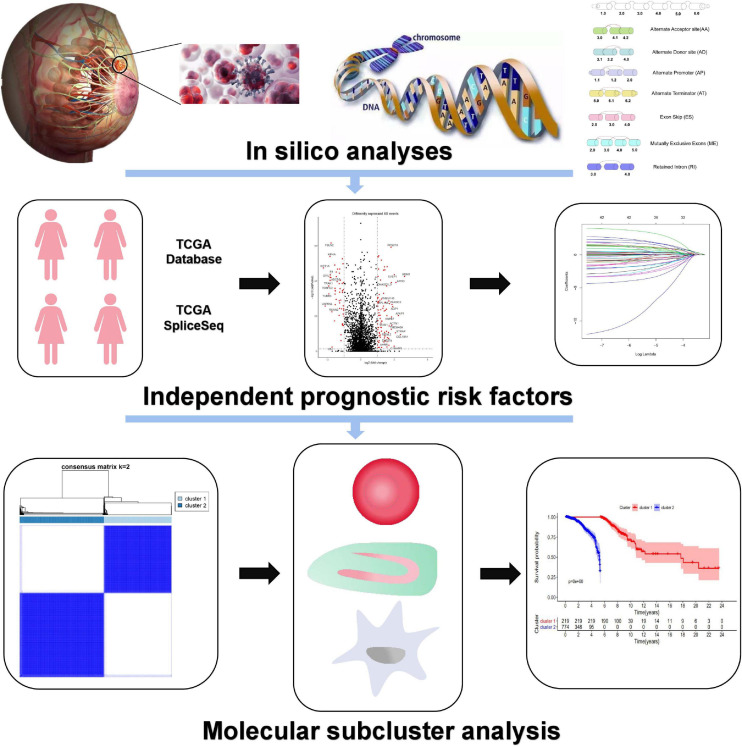
The flowchart for profiling AS of BrCa.

**FIGURE 2:**
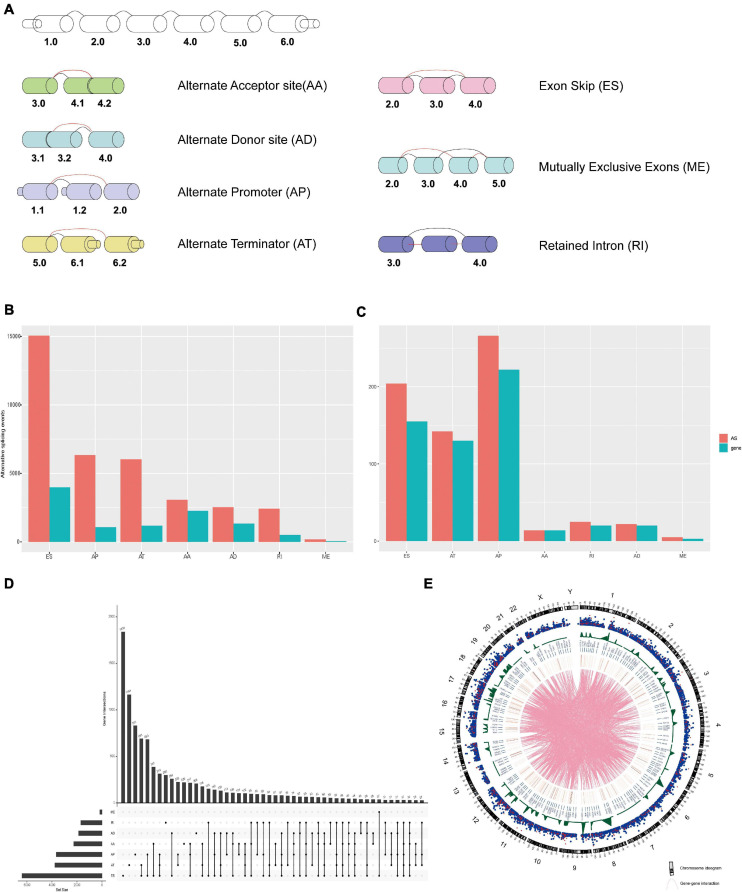
**(A)** The schematic diagram of the seven AS event patterns. **(B)** The number of AS events and their parental genes in BrCa patients. **(C)** The number of DEAS events and their parental genes in BrCa patients. **(D)** The upset diagram shows the intersection of the seven types of splicing events in BrCa. **(E)** Circos of AS events on chromosomes and their parent gene annotations. The outer circle consists of a point map representing the detected AS events, which is linked to the position of the parent gene in the chromosome, and the red dots represent the DEAS events. The blue dots represent 454 the non-differentially expressed AS events. The gene in the middle is called the DEAS event parent. 455 Lines represent potential interactions between parental genes of the DEAS events.

The flow diagram of the research is presented in Figure 1. A total of 993 patients who met the screening criteria were included in the study cohort, and the baseline characteristics of these patients are summarized in Supplementary Table 2. At a median follow-up of 30.2 months (range 1–286.8), 107 (10.8%) patients relapsed, and 136 (13.7%) patients died. The 5-year mortality and recurrence rates were 8.7 and 9.2%, respectively, with recurrences within 5 years accounting for 66.9% of the total deaths. We detected 35,520 AS events from 10,279 genes using rigorous filtering criteria. A total of 1,455,675 missing values were detected in the data integrity check, which accounted for approximately 4.1% of the total data volume. To maximize the statistical power of the data, we used multiple interpolation to estimate missing values. These AS events were classified into seven modes of splicing, including 3′ Alternate Acceptor site (AA), 5′ Alternate Donor site (AD), Alternate Promoter (AP), Alternate Terminator (AT), Exon Skip (ES), Mutually Exclusive Exons (ME), and Retained Intron (RI), as shown in Figure 2A. Among these splicing patterns, ES occurred most frequently (42.3%) (Figure 2B). A single gene may have multiple splicing patterns. Therefore, an upset map was created to analyze the interaction sets of seven types of AS events. Figure 2D shows that a single gene may have up to four different splicing patterns. A Circos diagram showed the position of AS events on the chromosome and the possible interactions between their parent genes (Figure 2E).

### Identification and Enrichment Analysis of DEAS Events

**FIGURE 3:**
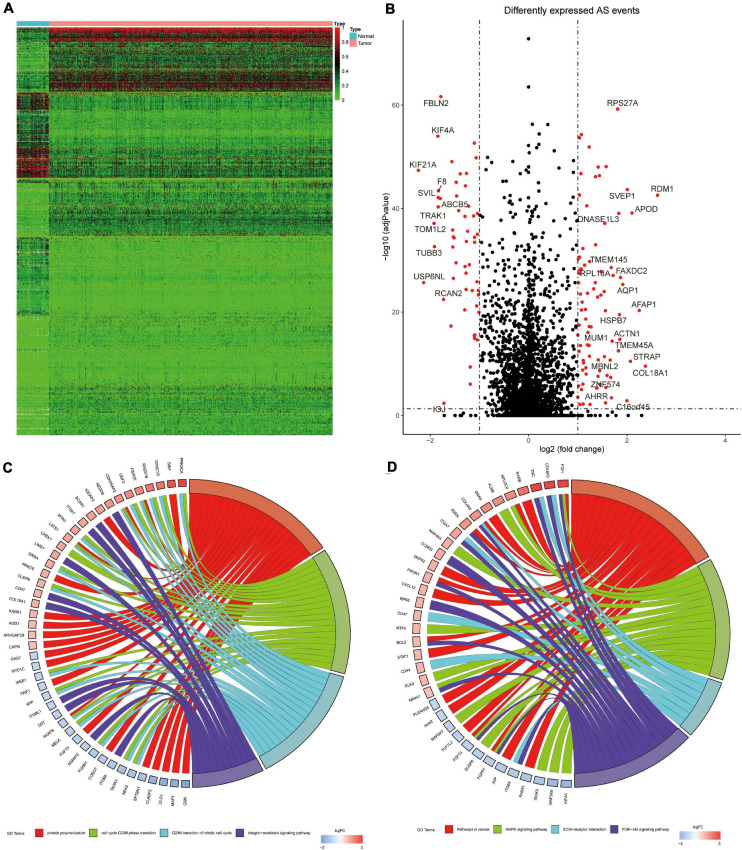
**(A)** Heat map of BrCa tissues and adjacent normal tissues. **(B)** Volcanic diagram of BrCa 458 DEAS events, with red dots representing low-expressed or high-expressed AS events. **(C)** The GO 459 analysis of BrCa DEAS event parent genes. **(D)** the KEGG analysis of BrCa DEAS event parent genes. In **(C,D)** the blue to red code next to the selected gene represents logFC, and the GO term represents the biological behavior related to the parent gene.

A total of 678 DEAS events of 564 genes were significantly different between 993 BrCa tissues and 113 adjacent normal tissues. Tumor and normal samples were clearly divided into two discrete groups using unsupervised hierarchical clustering based on DEAS events, which indicated that the screened DEAS events were credible (Figure 3A). The volcanogram showed high- and low-expressed DEAS events (Figure 3B). Notably, ES events had the highest frequency of all AS events, but the situation changed in DEAS events, and AP events accounted for the highest proportion (Figure 2C). The pattern of splicing was not evenly distributed, which suggests a different role in tumor progression. Abnormal AS events may directly affect the expression of its parent RNA.

The results showed that the parent genes of the DEAS events were enriched in pathways that were closely related to the BrCa process in GO analysis (Figure 3C). The pathways primarily included protein aggregation (FDR = 0.004), cell cycle G2/M phase transition (FDR = 0.028), G2/M transition mitotic cell cycle (FDR = 0.029), and integrin-mediated signaling pathways (FDR = 0.031). The KEGG pathways associated with BrCa tumorigenesis (Figure 3D) were enriched, such as the cancer pathway (FDR = 0.001), MAPK signaling pathway (FDR = 0.001), ECM-receptor interaction (FDR = 0.004), and PI3K-Akt signaling pathway (FDR = 0.005). Notably, the discovery of immune-related pathways, such as leukocyte migration across the endothelium (FDR = 0.002), aroused our interest, and we further examined the impact of variable splicing on tumor immunity.

### Construction of DEAS Events and SFs Regulatory Network

**FIGURE 4:**
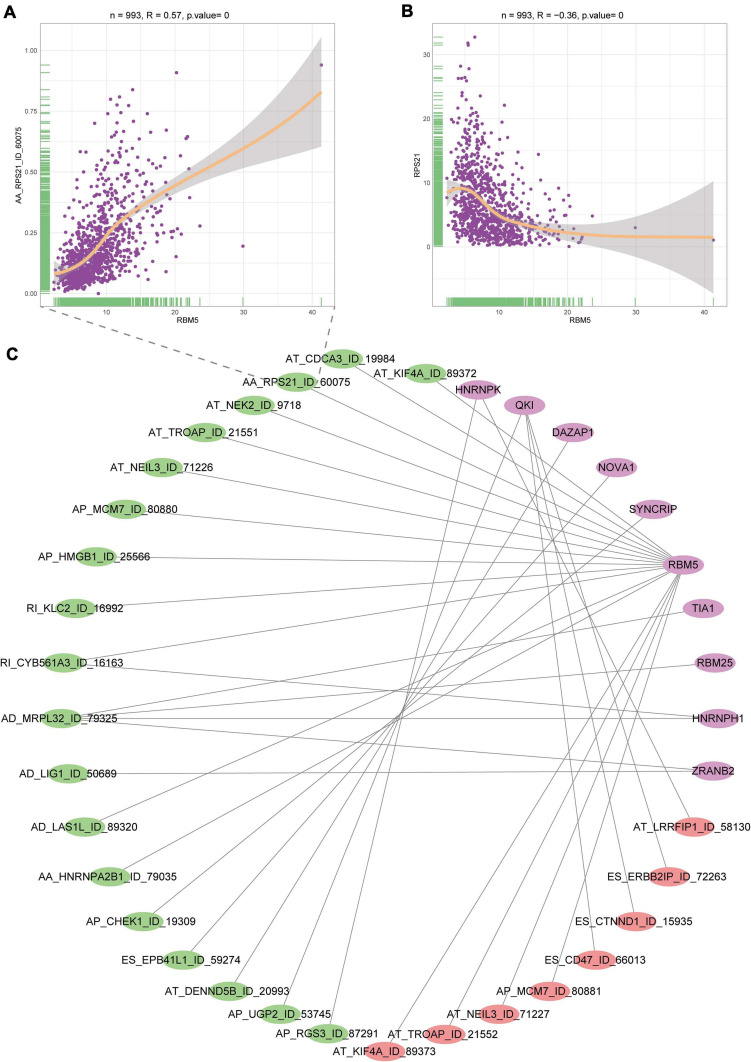
Interaction diagram between SFs and DEAS events. **(A)** Correlation diagram of the SF named RBMS and alternate splicing event of RPS21. **(B)** Correlation diagram of the SF named RBMS and parent gene expression of RPS21. **(C)** Network between SFs and AS events. Purple represents SFs, green represents positive correlation, and red represents negative correlation.

A splicing regulation network was established, and the expression of 10 SFs significantly correlated with the PSI of 27 DEAS events (including 19 positive correlations and 8 negative correlations). The network is shown in Figure 4C. Notably, four different SFs controlled a DEAS event, which reflects the complex cooperative and competitive relationships between SFs and partially explains the diversity of splice homologous patterns caused by only a few factors. For the AA splicing event of RPS21, its PSI value is positively correlated with the splicing factor (RBM5), but the expression of its parent gene is negatively correlated with the RBM5, which suggests that our previous mRNA detection methods ignore the diversity of gene transcripts (Figures 4A,B). Therefore, specific detection of gene transcripts can provide a more detailed description of tumor characteristics.

### Use of DEAS Events to Construct a Prognostic Risk Model

Univariate COX analysis showed that 48 and 49 DEAS events significantly correlated with OS and DFS, respectively. LASSO regression was used to filter variables and prevent over-fitting of the proportional hazards model (Supplementary Figure 1). These filtered DEAS events were included in the multivariate Cox regression analysis. 13 and 10 DEAS events were identified as independent prognostic factors for OS and DFS, respectively. Twenty-one independent prognostic risk factors were identified after the removal of repeats and were named total independent prognostic factors. The detailed results of these prognostic AS events are shown in the Supplementary Table 3.

### Classification, Prognosis, Molecular and Immune Characteristics Analysis Based on AS Events

**FIGURE 5:**
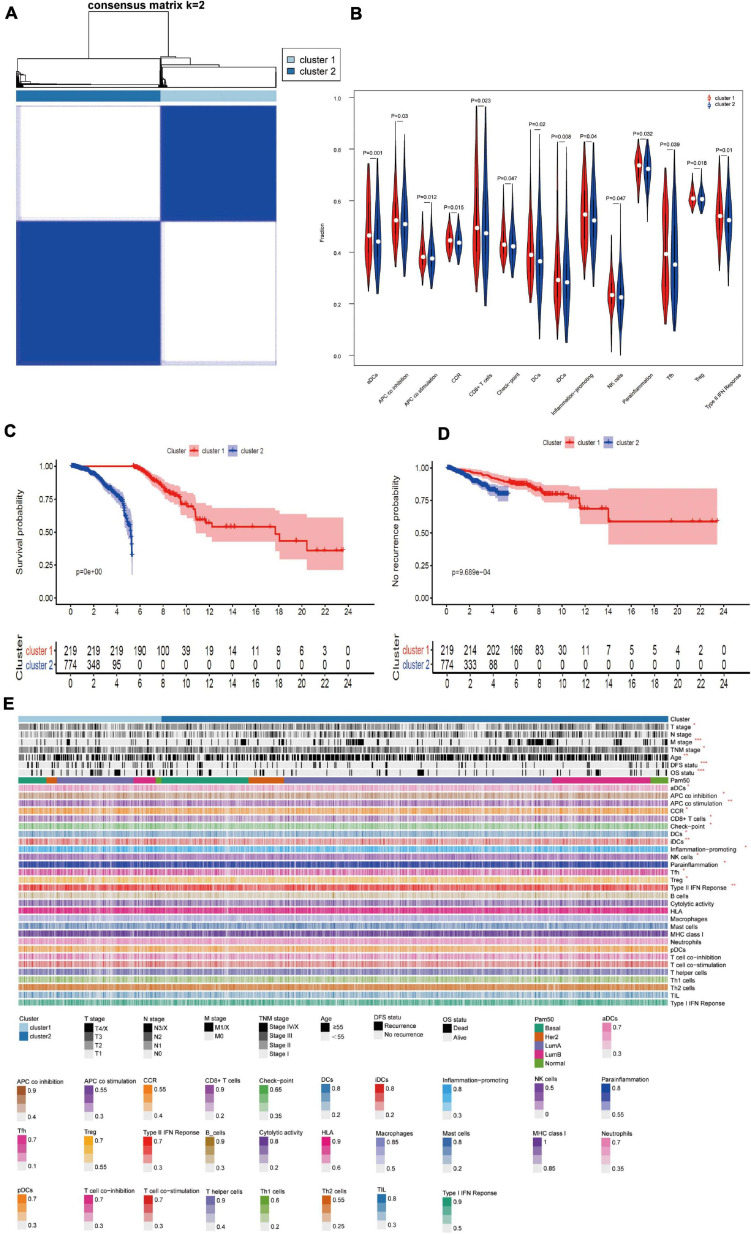
**(A)** Consensus cluster analysis identified two clusters (sample, *n* = 993). Heat maps of white (consensus = 0, samples never come together) and blue (consensus = 1, samples always come together) show sample consensus. **(B)** Violin diagram of immune-related components between; cluster 1 and 2. **(C,D)** Kaplan-Meier survival analysis for different molecular clusters based on OS or DFS; **(E)** Cluster analysis of clinicopathological information and immune components. (*P < 0.05 and ≤0.01; **P < 0.01 and ≤0.001; ***P < 0.001).

To understand the impact of independent prognostic AS events on patients, we performed an unsupervised cluster analysis. According to the consensus matrix heat map, patients were divided into two subclusters (Figure 5A): Cluster 1 (*n* = 219, 22.1%) and Cluster 2 (*n* = 774, 77.9%). We analyzed the clinicopathological characteristics and immune cell infiltration of the two subclusters. Kaplan-Meier survival analysis showed that the prognosis of Cluster 1 was significantly better than Cluster 2, regardless of OS or DFS (Figures 5C,D). As shown in the Figure 5E, T stage, M stage, TNM stage, age, and survival status (OS and DFS) were not randomly distributed between different clusters (*P* < 0.05). Immune cells in Cluster 1 were significantly higher than Cluster 2 (Figure 5B). These immune components included APC coinhibition, APC costimulation and CD8^+^ T cells, and these results are consistent with the previous conclusion that Cluster 1 has a better prognosis in OS and DFS. Briefly, the subgroup based on the independent prognostic AS events distinguished the immunophenotype and prognosis of BrCa patients, which indicates that this method has good clinical practical value. Figure 5E shows the cluster analysis of clinicopathological information and immune components. (^∗^ denotes *P* < 0.05 and ≥ 0.01; ^∗∗^ denotes *P* < 0.01 and ≥ 0.001; ^∗∗∗^ denotes *P* < 0.001).

### Real-Time Quantitative PCR Verification of DEAS Events in Tissues

To verify the accuracy of the bioinformatics analysis, we collected pairs of BrCa tissue and adjacent tissue samples for further verification. We reviewed studies of parental genes involved in all splicing events and selected one DEAS events for further validation. Two primers were designed for each gene to maintain consistency in the experimental approach. One primer was located on the splice sequence of the DEAS event, and the other primer was located on the CDS sequence of all transcripts. We used qPCR to obtain the splicing event and CD region expression of each gene, and the ratio of the two expression levels is the percent-spliced-in (PSI) value. A box diagram was generated to illustrate the qPCR results (Supplementary Figures 2A,B). The ratio of this DEAS event was significant upregulation in tumor tissue, which suggests that the increase in these DEAS events affects tumorigenesis. Notably, these findings provide important guidance for more detailed functional testing.

## Discussion

Current applications of targeted therapy are primarily focused at the gene level. However, studies at the post-transcriptional level found that the same gene had different functions. For example, Marina found that high expression of a new E-cadherin splice variant was associated with BrCa progression (Rosso et al., 2019). Catherine showed that the use of selective promoters leads to the overexpression of specific subtypes of ELF5, which may be a feature in the occurrence of BrCa (Piggin et al., 2016). Changes in splicing type lead to changes in the expression of certain AS events, which are of great significance in the occurrence and development of tumors (Chang and Lin, 2019).

Previous data on the function of BrCa AS events primarily focused on one or several genes, and there were no studies on the prognostic value of AS. The rapid development of high-throughput sequencing technology in recent years created favorable conditions for comprehensive discussions of the prognostic value of AS in BrCa.

Tumorigenesis of BrCa is a complex regulatory network. Compared to a single intuitive clinical indicator, the integration of multiple biomarkers into an aggregation model improves the ability of a model to predict prognosis. Over the past decade, great efforts were made to integrate genome-wide prognostic biomarkers to guide doctors in the early diagnosis and prognosis of BrCa patients. However, the focus of previous studies is limited to transcriptome level analyses and the mining of prognostic mRNA, lncRNA, or miRNA markers (Meng et al., 2014; Bing et al., 2016). Zhang et al. recently obtained RNA-seq data and corresponding clinical information of BrCa patients from the TCGA. The impact of AS events on the prognosis of patients was analyzed, and a survival prediction model was constructed (Zhang et al., 2019). Because the driving factors of tumorigenesis and development are generally differentially expressed in tumors and normal tissues (Meng et al., 2019), this article is limited because the data of adjacent normal tissues were not included in the study cohort. Therefore, we analyzed the differences in AS events between tumor tissues and adjacent normal tissues in our study and performed prognostic analyses based on DEAS events. Activation of immune-related pathways was found in functional enrichment analyses.

Twenty-one AS events were independent prognostic risk factors for OS or DFS. Several genes in these AS events play a vital role in tumor biology. For example, NFIB inhibits the growth of glioma cells, and its low expression may be an important reason for the occurrence of glioma (Li et al., 2019). Therefore, study of the functions and potential mechanisms of these AS events may be of great significance for the development of new therapeutic strategies. Due to the high degree of tumor heterogeneity, the clinical treatment of BrCa is significantly distinct in patients with different molecular subtypes. The present study identified two molecular clusters (Clusters 1, 2) based on total independent prognosis AS events. Stage T, stage M, stage TNM, age, and survival status (OS and DFS) were unevenly distributed between the molecular clusters. Notably, there were also significant differences in the infiltration of several important immune cells between the two groups, such as CD8^+^ T cells, NK cells and Treg cells. The content of CD8^+^ T and NK cells in Cluster 1 was higher than Cluster 2, which was also related to the good prognosis of OS and DFS. This conclusion is consistent with Peng GL, who suggested that CD8^+^ T cells were associated with a favorable prognosis in BrCa patients (Peng et al., 2019). NK cells are valuable cells in immunotherapy. The activation of these cells is driven by the balance between activation and inhibitory signals, and NKs exert anti-tumor functions without preactivation (Terrén et al., 2019). Treg cells participate in immunosuppression via a variety of mechanisms, and their high infiltration is related to poor survival in many of tumors. Our findings provide theoretical guidance for examining the relationships between alternative splicing and immunotherapy.

To examine the mechanisms of the effects of upstream factors on alternative splicing and the effects on immunity, we analyzed the relationships between 71 known SFs and splicing events. Our results showed that the abnormal expression of SF was closely related to the expression of DEAS events. A single SF may regulate multiple AS events, and an AS event may also be regulated by multiple SFs. Ribosomal protein s21 (RPS21) is a ribosome-related protein, which has been shown to play an important role in ribosomal biogenesis and may affect the occurrence and development of osteosarcoma and prostate cancer through the RAS/MAPK pathway (Arthurs et al., 2017; Fan et al., 2018; Liang et al., 2019; Sawyer et al., 2020; Wang et al., 2020). Through the query of the database GEPIA2, the expression of RPS21 gene is not significantly different in tumor and normal tissues (Supplementary Figure 2C), so its potential function in breast cancer has not been studied so far, but according to our research, one of the transcripts of RPS (AA- RPS21) is differentially expressed in cancer and normal tissues of breast cancer patients, which suggests that this transcript may play a tumor-driving role in breast cancer, so it is worthy of further investigation.

## Conclusion

In summary, the present study discovered that 21 independent prognostic AS events played important roles in the occurrence and development of breast cancer. These AS events may affect the prognosis and recurrence of patients via immune-related pathways, and we also found their potential upstream splicing mechanism. Our research provides directions for future research on the three levels of splicing factors, AS events and immune infiltration, and these new findings urgently need follow-up studies to support their clinical value.

## Data Availability Statement

The datasets presented in this study can be found in online repositories. The names of the repository/repositories and accession number(s) can be found in the article/Supplementary Material.

## Ethics Statement

This investigation was approved by the Ethics Committee of the Central Hospital of Xiangtan City. The patients/participants provided their written informed consent to participate in this study.

## Author Contributions

JW conceived and directed the project. YD designed the study and analyzed the data. YD, HZ, and LY wrote the manuscript. ZH and KF reviewed the data. All authors have read and approved the final manuscript for publication.

## Conflict of Interest

The authors declare that the research was conducted in the absence of any commercial or financial relationships that could be construed as a potential conflict of interest.

## Footnotes

1 https://portal.gdc.cancer.gov/
