# Supplementary figures and images for: Correlations Between the Characteristics of Alternative Splicing Events, Prognosis, and the Immune Microenvironment in Breast Cancer
Source: Front Genet. 2021 Jun 14;12:686298. doi: 10.3389/fgene.2021.686298 (PMC8236959; doi:10.3389/fgene.2021.686298)

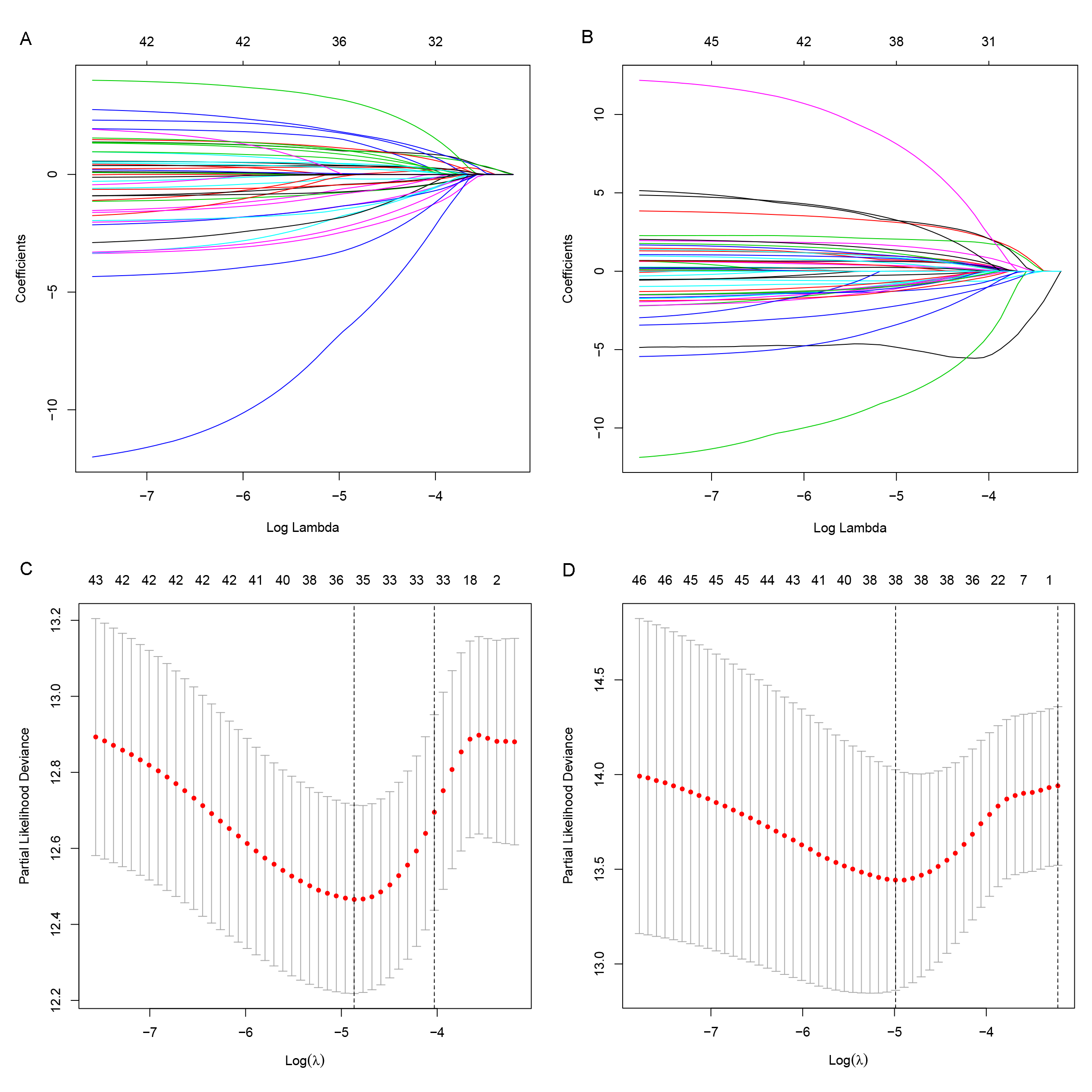

Supplement: Supplementary file 4 [file Image_1.TIF]
